# Interactions Between Child-Rearing and Other Risk Factors in Predicting Delinquency, and Implications for Prevention

**DOI:** 10.1177/0306624X231188231

**Published:** 2023-07-19

**Authors:** David P. Farrington, Catia G. Malvaso

**Affiliations:** 1 Cambridge University, UK; 2 University of Adelaide, SA, Australia

**Keywords:** delinquency, discipline, supervision, interaction, longitudinal

## Abstract

This article aims to identify interactions between harsh discipline and poor supervision and other childhood risk factors (all measured at age 8–10) in predicting delinquency. It analyzes data collected in the Cambridge Study in Delinquent Development (CSDD), which is a prospective longitudinal study of 411 London males first assessed at age 8. Of these males, 26% were convicted between ages 10 and 17. Harsh discipline and poor supervision significantly predicted delinquency, as did 16 other childhood risk factors. Generally, harsh discipline predicted delinquency more strongly in the presence of other risk factors, whereas poor supervision predicted delinquency more strongly in the absence of other risk factors. It is suggested that parent training programs targeting harsh discipline should focus particularly on children and families who possess other risk factors, whereas parent training programs targeting poor supervision should focus particularly on children and families who do not possess other risk factors.

## Introduction

The idea of risk-focused prevention is very simple (see e.g. Farrington, 2021b): Identify the key risk factors for delinquency, and implement programs that are designed to prevent or reduce their development. While numerous individual, familial, and broader contextual risk factors for delinquency have been identified (Farrington, 1995, 2007; Loeber & Dishion, 1983; Murray & Farrington, 2010), the focus of the present study is on better understanding the influence of two important child-rearing risk factors: harsh or erratic parental discipline and poor parental supervision. These risk factors have been selected because of their potential to be targeted in parent training programs, which numerous evaluation studies have shown can be effective in preventing delinquency (see e.g. De Vries et al., 2015). These programs aim to train parents to notice what a child is doing, monitor the child’s behavior over long periods, clearly state house rules, and make positive and negative reinforcements consistent and contingent on the child’s behavior. However, while a great deal is known about the most important childhood risk factors for delinquency, very little is known about interactions between risk factors, which can be very important. For example, if harsh discipline predicts delinquency only in low income families and not in higher income families, this suggests that (in the interests of implementing prevention programs most effectively) parent training should be particularly aimed at low income families.

The main aim of this article is to investigate interactions between the child-rearing factors of harsh discipline and poor parental supervision, and other important childhood risk factors, in predicting delinquency in the Cambridge Study in Delinquent Development (CSDD). The CSDD is a prospective longitudinal study of 411 London males, first assessed at age 8 in 1961 (see later). In a logistic regression analysis, Farrington and Malvaso (2019) found that harsh parental discipline was the strongest predictor of convictions for violence between ages 10 and 21, followed by a convicted father and low family income. Poor parental supervision was also a significant predictor, but it did not predict independently of the other three risk factors.

There have been several studies of interactions between risk factors in the CSDD. For example, Farrington and Bergstrom (2021) investigated interactions between risk factors at age 8 to 10 and resting heart rate at age 18 in predicting psychopathy at age 48. They found that a high resting heart rate acted as a protective factor in nullifying the effect of risk factors such as a convicted father and a depressed mother. Farrington and Ttofi (2011) found that good child-rearing acted as a protective factor against the risk factor of poor housing in predicting convictions up to age 50. The child-rearing variable was a combination of discipline and parental conflict. Farrington et al. (2016) completed the most extensive study of interactions between risk and protective factors at age 8 to 10 in predicting convictions between ages 10 and 18 inclusive. The most significant interaction effect was that high nonverbal intelligence acted as a protective factor against the risk factor of poor child-rearing.

This article aims to advance knowledge about interactions between harsh discipline and poor parental supervision and other important individual, family, and social risk factors for delinquency. There is a significant body of literature on the association between child maltreatment—including physical abuse and neglect—and delinquency that may provide insight into what is known about the interactions between these more serious forms of discipline and supervision and other risk factors in increasing the risk of delinquency and offending behavior. This literature is summarized next and has been used to inform the present study.

### Interactions Between Child Maltreatment and Other Risk Factors for Delinquency

Many aspects of parenting predict delinquency (see e.g. Hoeve et al., 2009). While the focus of the present study is on harsh parenting and poor parental supervision, it is acknowledged that at the most extreme end of these constructs are acts of commission or omission that constitute child maltreatment. There is mounting evidence from studies around the world that children who have been physically abused and/or neglected are at increased risk of subsequent criminal justice system contact compared to non-maltreated groups (Braga et al., 2017). Relatively fewer studies have explored interactions between abuse/neglect and other risk factors for offending behavior. These latter studies are important because the association between abuse/neglect and offending behavior is likely to be influenced by a combination of interacting stressors, risks, and protective factors (Malvaso et al., 2016). The risk of maladaptive behaviors, such as delinquency, is often conceptualized as multiply determined by individual characteristics (e.g., behavioral problems), the family or social context (e.g., harsh parenting), and the broader context (e.g., poverty; see Bronfenbrenner, 1979; Cicchetti & Toth, 1995). The central assumption is that these interacting stressors and risks shape delinquent behavior.

A handful of studies have also examined interactions between risk factors in the association between child abuse/neglect and delinquency. Some researchers have examined interactions between maltreatment and parental characteristics. In these studies, researchers have found that low parental education and high parental unemployment (Smith et al., 2005; Stouthamer-Loeber et al., 2001), as well as parental criminality (Widom & White, 1997), increase the likelihood of delinquency among maltreated children in adolescence. Topitzes et al. (2011) found that the risk of adolescent delinquency was increased among victims of child maltreatment who also had parents with low scholastic expectations of them in early childhood or who had low social-emotional skills in middle childhood (both as reported by teachers). This suggests that interventions that are designed to reduce child maltreatment and improve parenting practices, especially targeting disadvantaged populations, may be particularly effective in reducing delinquent behavior.

In terms of individual child characteristics, Smith et al. (2013) reported that maltreated children with higher grade-point averages and who had graduated from high school were less likely to be arrested when followed into early adulthood. Maltreated children with low cognitive functioning, poor social adjustment, and poor behavioral conduct were at increased risk of delinquency (Malvaso & Delfabbro, 2015; Taussig, 2002). An interesting finding from Taussig (2002) was that maltreated children with high levels of social acceptance from their classmates had a higher risk of engaging in delinquent behavior. It was suggested that this was perhaps due to associating with deviant peers, and that interventions focused on promoting the engagement of maltreated children with more prosocial peers might be effective in reducing delinquency.

In other studies, area level measures of disadvantage have been used to understand associations between child maltreatment and delinquency. For example, studies have demonstrated that the effect of child maltreatment on delinquency is stronger among socioeconomically disadvantaged populations (Bright & Jonson-Reid, 2008; Topitzes et al., 2011). Schuck and Widom (2005) demonstrated that neighborhood disadvantage and stability moderated the association between early experiences of abuse/neglect and both juvenile and adult arrests, with those from the most stable disadvantaged neighborhoods at increased risk of being arrested. It is important then to consider how interventions to reduce delinquency that are aimed at parents and/or their children might be influenced by both micro- and macro-level factors in families and communities.

While previous literature provides some insight into the possible interactions between child maltreatment and other child, parent and neighborhood characteristics in predicting delinquent behavior, most individual studies have only focused on one or two factors. The aim of this paper is to present a comprehensive, theoretically-driven approach to understanding different interactions, based on the major childhood risk factors measured in the CSDD.

### The Present Study

Understanding which variables interact in the prediction of delinquency, as well as the contexts within which associations between abuse/neglect and delinquency are exacerbated or mitigated, can provide key insights into opportunities for targeting prevention and early intervention initiatives. The present study aims to identify interactions between child-rearing factors, specifically harsh discipline and poor parental supervision, and other important childhood risk factors, in predicting delinquency.

It might be expected that risk factors would have additive effects in predicting delinquency. For example, consider the risk factors of low family income and harsh parental discipline. If 10% of children with neither risk factor became delinquent, 20% of those with only harsh discipline, 20% of those with only low income, and 40% of those with both low income and harsh discipline, this might indicate an additive effect of the two risk factors, but not an interaction effect. On the other hand, if 10% of those with neither risk factor became delinquent, 10% of those with only harsh discipline, 20% of those with only low income, and 50% of those with both risk factors, this might indicate an interactive effect of the two risk factors: harsh discipline increases the risk of delinquency in the presence of low income but not in the absence of low income. This effect, which might be termed an enhancing interaction effect, is the most common type of interaction effect that has been identified in previous research.

There is, however, another possible type of interaction effect that seems to have been overlooked previously. For example, if 10% of those with neither risk factor became delinquent, 30% of those with only harsh discipline, 40% of those with only low income, and 40% of those with both risk factors, this might also indicate an interaction effect: harsh discipline increases the risk of delinquency in the absence of low income, but not in the presence of low income. This might possibly be termed a suppressing interaction effect. The type of interaction effect that is found has important implications for prevention, because it indicates whether parent training is likely to be more effective in preventing delinquency in the presence of low income or in the absence of low income.

## Method

### Participants

The CSDD is a prospective longitudinal survey of 411 London males who were first studied in 1961 to 1962 at age 8 to 9. Their parents, teachers, peers, female partners, and children have also been interviewed. At the time they were first contacted in 1961 to 1962, the males were all living in a working-class area of South London. The vast majority of the sample was chosen by taking all the males who were then aged 8 to 9 and on the registers of six state primary schools within a one mile radius of a research office which had been established. Most boys were born in 1953. In addition to 399 males from these six schools, 12 males from a local school for “educationally subnormal” (special needs) children were included in the sample, in an attempt to make it more representative of the population of males living in the area. Therefore, the males were not a probability sample drawn from a population, but rather a complete population of males of that age in that area at that time.

Most of the males (357, or 87%) were White in appearance and of British origin, in the sense that they were being brought up by parents who had themselves been brought up in England, Scotland, or Wales. Of the remaining 54 males, 12 were African-Caribbean, having at least one parent of West Indian (usually) or African origin. Of the remaining 42 males of non-British origin, 14 had at least one parent from the North or South of Ireland, 12 had parents from Cyprus, and the other 16 males were White and had at least one parent from another Western industrialized country.

On the basis of their fathers’ occupations when they were aged 8, 94% of the males could be described as working-class (categories III, IV, or V on the Registrar General’s scale, describing skilled, semi-skilled, or unskilled manual workers), in comparison with the national figure of 78% at that time. The majority of the males were living in conventional two-parent families with both a father and a mother figure; at age 8, only 6% of the males had no operative father and only 1% had no operative mother. This was, therefore, overwhelmingly a traditional White, urban, working class sample of British origin.

The males have been interviewed nine times, at ages 8, 10, 14, 16, 18, 21, 25, 32, and 48. At all ages except 21 and 25, the aim was to interview all the males who were still alive, and it was always possible to interview a high proportion: 405 (99%) at age 14, 399 (97%) at age 16, 389 (95%) at age 18, 378 (94%) at age 32, and 365 (93%) at age 48.

The results of the CSDD have been described in six books (Farrington et al., 2013; Piquero et al., 2007; West, 1969, 1982; West & Farrington, 1973, 1977), and in nine summary articles (Farrington, 1995, 2003, 2019a, 2021a; Farrington & Jolliffe, 2022; Farrington et al., 2009, 2021; Farrington & West, 1981, 1990).

As mentioned, the main aim of this article is to investigate interactions between child-rearing factors and other childhood risk factors (measured at age 8–10) in predicting delinquency.

#### Delinquency

Delinquency was defined according to convictions for offenses committed between ages 10 and 17 inclusive. Information about convictions was obtained from repeated criminal record searches beginning in 1964. Only relatively serious offenses were recorded, for example, excluding almost all motoring offenses, and only crimes committed on different days were counted, so that each offense arose from a different criminal incident. The most common crimes were burglary, theft, violence, vandalism, fraud, and drug use (Farrington, 2019b).

In a chapter that was mainly concerned with young adult offending between ages 18 and 25, Farrington (2012) reported that 103 boys were convicted for offenses committed between ages 10 and 17 inclusive, and summarized the extent to which key childhood (age 8–10) risk factors predicted them. Three boys were excluded from the analyses, because they emigrated permanently before age 17 (two at age 9 and one at age 14). In the present article, two other boys who were convicted at age 8 (before the minimum age for conviction was increased from 8 to 10 in England in February 1964) were counted as delinquents. Therefore, 105 boys out of 408 (25.7%) are counted as delinquents in the present article.

#### Age 8 to 10 Variables

The two main child-rearing factors that were measured in the CSDD were harsh parental discipline and poor parental supervision. These were based on ratings by psychiatric social workers who visited the boys’ parents. The harsh discipline measure included erratic discipline and a cold parental attitude, while poor parental supervision referred to the extent to which the parents knew what the boy was doing when he was outside the house.

Sixteen other childhood risk factors that predicted delinquency were extracted from Farrington (2012). Convictions of a boy’s biological parent, up to the boy’s tenth birthday, were obtained from criminal record searches. A young mother referred to a mother who was a teenager at the time of her first birth. Parental conflict, also rated by the psychiatric social workers, referred to chronic tension or disagreement in many fields, or raging conflicts. A disrupted family referred to the temporary or permanent separation of the boy from a parent (usually the father) before the boy’s tenth birthday, for reasons other than death or hospitalization. Low family income and poor housing (very dilapidated) were rated by the psychiatric social workers. Whereas every other risk factor identified between 19 and 29% in the risk category, 37% of the boys were in poor housing. Large family size referred to five or more children born to the boy’s mother before his tenth birthday. Low socioeconomic status referred to the family breadwinner (usually the father) having an unskilled manual job. Information about high delinquency-rate schools was obtained from the local authority.

Low nonverbal intelligence of the boy was measured by the Progressive Matrices tests that were given to the boys in schools, while low verbal intelligence was based on verbal comprehension and vocabulary tests. Low junior school attainment of the boy was based on school records of arithmetic, English and verbal reasoning tests completed by the boys. High daring was rated by parents and peers, and identified boys who took many risks in traffic, climbing, exploring, and so on. High hyperactivity was based on ratings by teachers of whether the boy lacked concentration or was restless in class. High troublesomeness was rated by peers and teachers, identifying boys who got into trouble most, and high dishonesty was rated by peers.

These childhood risk factors were measured in 1961 to 1962. They were chosen by Donald West, who initiated the CSDD, based on the best available knowledge at that time about the causes and predictors of delinquency. He was particularly inspired by the work of McCord et al. (1959; see West, 1969, 1982), and Joan McCord gave him a great deal of detailed information about the coding and definition of variables such as discipline and supervision, together with numerous examples of codings such as “harsh discipline” and “poor supervision.” Many age 8 to 10 variables were closely modeled on the variables measured by Joan McCord in the Cambridge-Somerville Youth Study.

#### Analytic Strategy

In the present article, as in many CSDD analyses, the age 8 to 10 variables were dichotomized into the “worst” quarter (the risk category) versus the remainder (the nonrisk category). This facilitated a “risk factor” approach, made all the risk factors comparable, and did not usually involve much loss of information, as many variables were originally measured on 2, 3, or 4 point scales (Farrington & Loeber, 2000). Most variables were not measured on normally distributed equal-interval scales. Most importantly for the present article, dichotomized predictors and outcomes permit an easy and very understandable method of studying interaction effects, compared for example with multiplying continuous variables that may not be measured on equal interval scales, may not be normally distributed, and may not be linearly related.

The odds ratio (*OR*) effect size was used to measure the strength of relationships, and interaction effects were assessed using analysis of variance. In concordance with the American Statistical association (e.g. Wasserstein & Lazar, 2016) and editorial statements from journals (e.g. Sullivan & Feinn, 2012; Trafimow & Marks, 2015), we believe that effect sizes are more important than p-values in indicating the strength of relationships (e.g. because *p*-values are highly dependent on sample size). Therefore, we focus on effect sizes. As pointed out by Cohen (1996, p. 136), “ the field of epidemiology tends to regard *OR* of 2.0 or more as fairly large.”

## Results

Table 1 shows the extent to which the 18 childhood risk factors predicted delinquency. For example, 42 out of 114 boys (36.8%) who were receiving harsh parental discipline were convicted, compared with 55 out of 274 other boys (20.1%) who were known on this variable (*OR* = 2.32, *z* = 3.41, *p* = .0003; one-tailed *p*-values are used here in light of the clear directional predictions). As another example, 30 out of 73 boys (41.1%) who were receiving poor supervision were convicted, compared with 65 out of 307 other boys (21.2%) who were known on this variable (*OR* = 2.60, *z* = 3.46, *p* = .0003).

**Table 1.** Relations With Delinquency, Harsh Discipline, and Poor Supervision.

Risk factor	Delinquency			Discipline	Supervision
	% NR	% R	*OR*	*OR*	*OR*
Child-rearing					
Harsh discipline	20.1	36.8	2.32*	—	4.00*
Poor supervision	21.2	41.1	2.60*	4.00*	—
Family					
Convicted parent	17.7	47.7	4.23*	1.94*	3.18*
Young mother	22.2	38	2.16*	1.52*	2.03*
Parental conflict	20.6	36.4	2.21*	5.93*	3.56*
Disrupted family	21.7	40	2.41*	1.84*	2.24*
Socioeconomic					
Low family income	21	41.9	2.72*	2.14*	5.60*
Poor housing	20.5	34.7	2.05*	1.49*	3.16*
Large family size	20.1	43.4	3.06*	1.55*	3.65*
Low SES	23.7	34.2	1.67*	1.74*	3.20*
Delinquent school	22.3	45.5	2.91*	1.62*	3.25*
Attainment					
Low nonverbal IQ	21.9	37.3	2.12*	1.85*	2.92*
Low verbal IQ	23	34.7	1.77*	1.14	3.06*
Low attainment	20.4	41.1	2.72*	1.67*	1.54
Individual					
High daring	17.6	45.5	3.90*	1.99*	3.26*
High hyperactivity	22.4	39.5	2.26*	1.97*	2.08*
High troublesomeness	18.3	51.6	4.77*	2.25*	4.07*
High dishonesty	18.9	42	3.11*	1.91*	2.91*

*Note*. SES = Socioeconomic Status; NR = Nonrisk category; R = Risk category; *OR* = Odds Ratio.

* *p* < .05, one-tailed.

Regarding the other risk factors, the strongest predictors of delinquency were high troublesomeness (*OR* = 4.77) and a convicted parent (*OR* = 4.23). It is not surprising that high troublesomeness was the strongest predictor, because it is probably measuring the same underlying construct (e.g. antisocial potential; see Farrington, 2020) as delinquency. In contrast, a convicted parent is clearly measuring a different underlying construct from delinquency, and therefore is a possible causal factor. The weakest predictor was low SES (socioeconomic status), possibly because some manual jobs (e.g. as printers and dockers) were quite well paid and sought after in the early 1960s in London. However, every one of these 18 predictive relationships was statistically significant, and 16 of them were greater than *OR* = 2.0.

Table 1 also summarizes the relation between the two child-rearing factors and the other 16 risk factors. Harsh discipline and poor supervision were significantly inter-related (*OR* = 4.00). Harsh discipline was most strongly related to parental conflict (*OR* = 5.93), while poor supervision was most strongly related to low family income (*OR* = 5.60). Every relationship was statistically significant except for low verbal IQ versus harsh discipline and low attainment versus poor supervision. However, whereas 15 out of 16 relationships with poor supervision were greater than *OR* = 2.0, this was true of only 3 out of 16 relationships with harsh discipline.

Table 2 shows the extent to which harsh discipline predicted delinquency in risk and nonrisk categories. For example, in low income families, 24 out of 38 boys (63.2%) receiving harsh discipline were convicted, compared with 12 out of 51 other boys (23.5%; *OR* = 5.57, *z* = 3.64, *p* = .0001). In contrast, in higher income families, 18 out of 76 boys (23.7%) receiving harsh discipline were convicted, compared with 43 out of 223 other boys (19.3%; *OR* = 1.30, *z* = 0.83, not significant). There was a significant interaction between harsh discipline and low family income in predicting delinquency (*F* = 11.28, *p* = .001). Clearly, harsh discipline predicted delinquency much more in low income families than in higher income families.

**Table 2.** Harsh Discipline Versus Delinquency in Risk and Nonrisk Categories.

Risk factor	Nonrisk			Risk			Interaction	
	% NH	% H	*OR*	% NH	% H	*OR*	*F*	*p*
Family								
Convicted parent	15.2	21.1	1.49	35.9	62.8	3.01*	4.59	.033
Young mother	18.3	31.7	2.08*	27.3	50	2.67*	0.72	.396
Parental conflict	18.2	30	1.93*	24.2	43.4	2.40*	0.43	.513
Disrupted family	16.8	30.8	2.20*	33.3	50	2	0.07	.799
Socioeconomic								
Low family income	19.3	23.7	1.3	23.5	63.2	5.57*	11.28	.001
Poor housing	16.8	26.6	1.80*	26.3	50	2.80*	2.09	.149
Large family size	15.6	28.2	2.13*	35.5	55.6	2.27*	0.52	.472
Low SES	19.3	33.3	2.09*	23.9	46.7	2.78*	0.58	.445
Delinquent school	18	31.6	2.10*	37.8	53.6	1.9	0.04	.852
Attainment								
Low nonverbal IQ	19.4	25.3	1.41	22.4	59	4.98*	8.79	.003
Low verbal IQ	19.4	31	1.86*	23.1	53.3	3.81*	2.95	.087
Low attainment	16.8	27.8	1.90*	35.8	48.4	1.68	0.02	.886
Individual								
High daring	16	22.1	1.48	32.8	60	3.07*	4.63	.032
High hyperactivity	20	28.4	1.59	20.5	59.4	5.68*	7.41	.007
High troublesomeness	16.8	21.1	1.32	35.4	68.4	3.95*	7.74	.006
High dishonesty	16.4	21.7	1.41	36.5	50	1.74	0.54	.462

*Note. F* test based on Analysis of Variance (two-tailed *p* values). SES = Socioeconomic Status; NH = Nonharsh category; H = Harsh Category; *OR* = Odds Ratio.

* *p* < .05, one-tailed.

There were also significant interaction effects between harsh discipline and a convicted parent, low nonverbal intelligence, high daring, high hyperactivity, and high troublesomeness. The number of significant interaction effects (6 out of 16) was significantly greater than chance expectation (mean = 0.80, standard deviation 0.87). In each case, harsh discipline predicted delinquency significantly in the risk category, but not in the nonrisk category. These were clearly enhancing interaction effects. In 13 out of 16 cases, harsh discipline predicted delinquency more strongly in the risk category than in the nonrisk category.

Table 3 shows similar analyses for poor parental supervision. However, the results are very different from harsh discipline. In 14 out of 16 cases, poor supervision predicted delinquency more strongly in the nonrisk category than in the risk category. For example, in low SES families, 9 out of 27 boys (33.3%) receiving poor supervision were convicted, compared with 15 out of 47 other boys (31.9%; *OR* = 1.07, *z* = 0.13, not significant). In contrast, in higher SES families, 21 out of 46 boys (45.7%) receiving poor supervision were convicted, compared with 50 out of 260 other boys (19.2%; *OR* = 3.53, *z* = 3.76, *p* = .0001). There was a significant interaction between poor supervision and a low SES family in predicting delinquency (*F* = 4.12, *p* = .043). This was clearly a suppressing interaction effect. However, this was the only significant interaction in Table 3, although poor supervision almost significantly interacted with a high delinquency-rate school (*F* = 3.03, *p* = .083). The number of significant interaction effects (one out of 16) was not significantly greater than the chance expectation of 0.80.

**Table 3.** Poor Supervision Versus Delinquency in Risk and Nonrisk Categories.

Risk factor	Nonrisk			Risk			Interaction	
	% NP	% P	*OR*	% NP	% P	*OR*	*F*	*p*
Family								
Convicted parent	15.1	28.9	2.30*	42.6	54.3	1.6	0.04	.841
Young mother	18.5	38.8	2.78*	32.2	45.8	1.78	0.29	.591
Parental conflict	18.6	35	2.35*	29.4	46.9	2.12	0.01	.926
Disrupted family	17.2	37	2.82*	36.5	48.1	1.61	0.47	.494
Socioeconomic								
Low family income	19.8	28.6	1.62	28.6	52.6	2.78*	1.64	.202
Poor housing	17.6	34.5	2.46*	28.9	45.5	2.05*	0	.981
Large family size	17.1	31.6	2.24*	37.7	51.4	1.75	0.01	.946
Low SES	19.2	45.7	3.53*	31.9	33.3	1.07	4.12	.043
Delinquent school	17.8	43.9	3.61*	42.2	46.2	1.17	3.03	.083
Attainment								
Low nonverbal IQ	18.5	33.3	2.20*	31.3	51.6	2.35*	0.23	.635
Low verbal IQ	20.1	40.5	2.71*	26.7	41.9	1.99	0.19	.667
Low attainment	16.5	37.8	3.07*	37.1	47.4	1.53	0.72	.395
Individual								
High daring	15.5	30.6	2.41*	40.3	51.4	1.57	0.13	.72
High hyperactivity	18.9	38.5	2.68*	32.7	47.6	1.87	0.13	.717
High troublesomeness	16	29.3	2.17*	47.1	56.3	1.45	0.12	.726
High dishonesty	15.6	34.4	2.83*	36.8	48	1.58	0.36	.548

*Note. F* test based on Analysis of Variance (two-tailed, *p* values). SES = Socioeconomic Status; NP = Nonpoor category; P = Poor Category; *OR* = Odds Ratio.

* *p* < .05, one-tailed.

## Discussion

The results of this research show that harsh discipline especially predicts delinquency in the presence of other risk factors, particularly a convicted parent, low family income, low nonverbal intelligence, high daring, high hyperactivity, and high troublesomeness. These findings are concordant with previous research on child maltreatment versus delinquency that was reviewed in the Introduction; the effects of child maltreatment on delinquency were stronger in economically disadvantaged populations, among children with convicted parents, and among badly behaved children. A major implication of these results is that theories of the development of delinquency should take account of interaction effects; in general, they just specify the additive effects of risk factors (see e.g. Farrington, 2006).

The most surprising findings on harsh discipline versus delinquency were that harsh discipline was more strongly predictive in the absence of a disrupted family, a high delinquency-rate school, and low school attainment. Further research would be needed to try to explain these deviant results.

Nevertheless, it follows that parent training programs that target harsh discipline would have more effect in reducing delinquency if they targeted children and families with the first set of risk factors. For example, if an effective parent training program eliminated the problem of harsh discipline in low income families, that might reduce the prevalence of delinquency of children from 63.2 to 23.5%, a relative reduction of 63%. However, if this program eliminated the problem in higher income families, this might reduce the prevalence of delinquency from 23.7 to 19.3%, a relative reduction of only 19%.

The same conclusions, however, do not follow for programs that tackle poor parental supervision. In almost all cases, poor supervision predicted delinquency less strongly in the presence of other risk factors than in the absence of other risk factors. Therefore, parent training programs that target poor supervision may have more effect in reducing delinquency if they targeted children and families who do not possess these risk factors. The main exception to this statement is low family income, since poor supervision predicted delinquency more strongly in the presence of low family income than in its absence. Again, further research would be needed to investigate why low family income produced a deviant result in this case.

These results are, of course, based on small numbers in one longitudinal study. The present study, like all studies, has limitations. In particular, the age 8 to 10 variables were measured 60 years ago, and the sample was predominantly a traditional two-parent White working-class urban male sample of British origin. The extent to which the results can be generalized to the present day or to other populations (e.g. single-parent, non-White, middle-class, rural, female, non-British) can only be determined by further research. However, careful comparisons of risk factors in the CSDD with risk factors in more recent longitudinal projects such as the Pittsburgh Youth Study (Farrington & Loeber, 1999) and the Zurich Z-Proso study (Zych et al., 2021) have discovered many similarities in results (see also Farrington, 2015).

Ideally, several other longitudinal studies should be analyzed to investigate interactions between child-rearing factors and other childhood risk factors in predicting delinquency, in order to establish the most important and most replicable interaction effects. Also, in evaluating the impact of parent training programs, more efforts should be made to investigate childhood risk factors that influence the success of these programs, in the interests of implementing parent training programs more efficiently. Also, similar analyses should be carried out for other risk factors that are targeted in intervention programs. For example, the risk factors of high impulsiveness and low self-control are targeted in child skills training programs (Beelman & Lösel, 2021). It would be desirable to know to what extent these risk factors interacted with other childhood risk factors in predicting delinquency, and to what extent the success of these programs varied according to the presence or absence of these other risk factors.

### Parenting Interventions

Child-rearing factors, such as harsh discipline and abuse/neglect, have influence in the context of family and broader social factors. Therefore, parenting interventions designed through an ecological lens may be more effective in reducing undesirable outcomes later in life, such as delinquency (Melton, 1992; Schuck & Widom, 2005; Widom, 2000). It is also known that intervening with both the child and the family early in the developmental pathway can reduce later delinquent outcomes (Farrington & Welsh, 2002). A series of evaluations of programs such as the Elmira Prenatal/Early Infancy Project (Eckenrode et al., 2010; Olds, 2002), the Perry Preschool Project (Schweinhart, 2013), and the Seattle Social Development Project (Hawkins et al., 1999) have demonstrated desirable effects on academic achievement and family wellbeing, and a reduction in behavioral problems and delinquent behavior, particularly for children from disadvantaged backgrounds.

A number of meta-analytic reviews have demonstrated desirable effects of early developmental prevention programs in reducing delinquent outcomes. In a meta-analytic review, Farrington and Welsh (2003, 2007) found that programs targeting family risk factors such as poor child-rearing, including harsh or inconsistent discipline and poor supervision, were effective in reducing delinquency. While school-based programs tended to be the least effective, home-visiting, preschool and multi-systemic therapy programs tended to be the most effective. A more recent meta-analysis of early parent training programs found that these were effective in reducing early behavioral problems in childhood, with some evidence that this effectiveness extended to delinquent behaviors in adolescence and early adulthood (Piquero et al., 2009, 2016). Consistent with these findings, in a meta-analysis examining the effectiveness of early developmental prevention programs delivered to children up to age 5 who were identified as at-risk for non-health outcomes during adolescence (including delinquency), Manning et al. (2010) reported a mean effect size (Cohen’s *d*) of 0.48 for outcomes relating to deviance, but a smaller mean effect size of 0.24 for outcomes relating to criminal justice system involvement. Taken together, these findings indicate that early developmental prevention programs may play an important role in crime reduction policy (see also Farrington, 2021b; Welsh et al., 2022).

Despite this, some recent reviews have cast some doubt on the quality of the evidence upon which conclusions about the effectiveness of such programs have been drawn. For example, Lynch (2017) examined the evidence from randomized and quasi-experimental studies of some of the most well-known early-life child health and development programs, such as the Nurse Family Partnership program. While there were consistent desirable effects across trials for a number of outcomes, many of the trials were not large enough to examine subgroup effects among different population groups (e.g., mothers with mental health problems). Lynch (2017) concluded that these trials were underpowered and that the data should not be used to reliably estimate effect sizes within different population subgroups. These conclusions were consistent with the review of Home Visiting Programs by the US Department of Health and Human Services (OPRE, 2016), with less than half of the research on these evaluations assessed as being of moderate to high quality.

Generally, there is a need for better quality evidence to understand the effects of programs on complex social outcomes, such as delinquency, that are likely to be caused by a complex interplay of risk and protective factors. The present study suggests that harsh discipline predicts delinquency more strongly in the presence of other risk factors, and this finding might assist in targeting intervention efforts. However, poor parental supervision predicts delinquency more strongly in the absence of other risk factors. It is clear that further high-quality research is needed to understand which factors should be targeted, when, for whom, and in what contexts, in order to improve outcomes for children at risk of delinquency.
